# Milk Protein Concentration Using Negatively Charged Ultrafiltration Membranes

**DOI:** 10.3390/foods7090134

**Published:** 2018-08-28

**Authors:** Abhiram Arunkumar, Mark R. Etzel

**Affiliations:** Department of Chemical and Biological Engineering, University of Wisconsin, 1605 Linden Drive, Madison, WI 53706, USA; arunkumar@uwalumni.com

**Keywords:** dairy, casein, deposit layer, retentate, permeate, polyethersulfone, regenerated cellulose, sieving coefficient

## Abstract

In this work, milk protein concentrate (MPC) was made using wide-pore negatively charged ultrafiltration membranes. The charged membranes were used for a six-fold volume concentration of skim milk and subsequent diafiltration to mimic the industrial MPC process. The charged 100 kDa membranes had at least a four-fold higher permeate flux at the same protein recovery as unmodified 30 kDa membranes, which are currently used in the dairy industry to make MPC. By placing a negative charge on the surface of an ultrafiltration membrane, the negatively charged proteins were rejected by electrostatic repulsion and not simply size-based sieving. Mass balance models of concentration and diafiltration were developed and the calculations matched the experimental observations. This is the first study to use wide-pore charged tangential-flow membranes for MPC manufacturing. Additionally, a unique mass balance model was applied, which accurately predicted experimental results.

## 1. Introduction

Dairy protein ingredients are ubiquitous in snack foods and infant formula, foods for the elderly, and foods for fitness enthusiasts. Specific milk proteins like β-casein, alpha-lactalbumin (ALA), lactoferrin and lactoperoxidase are used in humanized infant formula [1]. Micellar casein concentrate is used in cheese manufacturing. The properties and uses of different milk proteins can be found elsewhere [2,3,4,5,6,7,8].

Skim milk contains approximately 8.6% in total solids, of which proteins account for 3.2% and lactose, ash, non-protein nitrogen (NPN) and small molecules comprise the other 5.4% [8]. The remainder is water. On a dry basis, skim milk consists of approximately 35–37% protein. The milk protein is grouped into casein and the serum proteins, which range in molecular mass from 14.4–150 kDa [3]. The purpose of milk protein concentration is to increase the protein-to-dry-solids content from about 37% to 80% (or higher) by removing water and the other small molecules while retaining protein. This is accomplished using ultrafiltration.

Industrial practice is to produce milk protein concentrate (MPC) using ultrafiltration membranes that have a nominal molecular weight cut-off (MWCO) ranging from 10 kDa to 30 kDa [9,10,11]. These tight membranes retain proteins and allow lactose and small molecules to permeate. However, tight membranes have a low hydraulic permeability and permeate flux. Wider pore size membranes increase the hydraulic permeability, but at the expense of higher protein losses. For example, Holland et al. [12] found that ALA permeated a 100 kDa unmodified ultrafiltration membrane while most other proteins were retained. Piry et al. [13] reported a greater than 50% transmission of beta-lactoglobulin (BLG) through a 0.1 μm microfiltration membrane, although it could operate at high permeate fluxes.

The purpose of the present research was to examine the flux enhancement in MPC manufacturing using wide-pore-size, negatively charged, tangential flow ultrafiltration membranes and characterize membrane performance for a six-fold concentration and diafiltration process. As the major milk proteins have an average isoelectric point (pI) of 4.6, the proteins have a net negative charge at the neutral pH of milk (pH 6.7), and are electrostatically rejected by a wide-pore size negatively charged membrane. Due to the wider pores, these membranes allow operation at a higher hydraulic permeability and permeate flux then narrow pore membranes. An additional purpose of this work was to develop accurate mass balance models of the MPC manufacturing process to replace costly and time-consuming trial-and-error experimentation in the design and optimization of new processes.

The ultrafiltration of milk is different from our previous work on whey protein concentrate [14]. When whey proteins are concentrated, there is not as significant an increase in viscosity because the predominant whey proteins, ALA and BLG, are small proteins with molecular masses of 14.4 and 18.4 kDa and hydrodynamic diameters of 1.8 and 2.7 nm, respectively [15].

In contrast, the predominant proteins in skim milk are caseins, which, being micellar in nature, have an effective hydrodynamic diameter of 165 nm [13], and are completely retained by ultrafiltration membranes at 22 °C. The caseins form a highly negatively charged deposit layer at the surface of the membrane [11,16]. The main reason for falling permeate flux in ultrafiltration membranes is the electrostatic interactions in the casein deposit layer [16]. As skim milk is concentrated, the viscosity of the retentate increases due to the concentration of the casein micelles and causes a significant increase of viscosity over a six-fold volume concentration [17]. This leads to a high retentate pressure drop, low tangential shear rate, and severe flux decline in constant pressure operations, creating a thicker deposit layer. One method to overcome these limitations is to decouple the pressure drop and the tangential shear rate by using rotating disk filtration hardware [17]. The present work controlled the permeate flux instead of the pressure drop to prevent the formation of a thick deposit layer. This was a key difference in realizing the benefits of wide-pore size, negatively charged ultrafiltration membranes for milk protein concentration.

## 2. Theory

### Mass Balance Equations for Ultrafiltration and Diafiltration

Protein transmission is characterized by the observed sieving coefficient, *S_O_* = *C_P_*/*C_R_*, where *C_P_* and *C_R_* are the instantaneous concentrations of protein in the permeate and retentate, respectively. A “mixing cup” concentration, denoted by brackets <*C_P_*> or <*C_R_*>, is what one would measure if the liquid issuing from a tube was collected in a cup and thoroughly mixed. A unique aspect of the present mass balance model is that it was not assumed that *C_R_* = <*C_R_*> as in past models.

**Figure 1:**
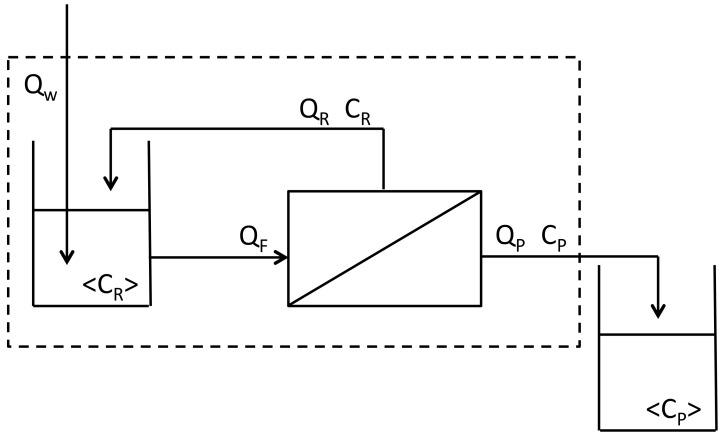
Flow system for ultrafiltration and constant volume diafiltration. Flow rates of diafiltration water (*Q_W_*), permeate (*Q_P_*), retentate (*Q_P_*), and feed solution (*Q_F_*). Instantaneous concentrations of permeate (*C_P_*) and retentate (*C_P_*). Mixing-cup concentrations of permeate (<*C_P_*>) and retentate (<*C_P_*>).

A single-stage ultrafiltration/diafiltration system is shown in Figure 1. The feed tank contains skim milk of concentration *C_F_* and volume *V_F_* at time zero. The volume in the feed tank at any later time is *V_R_*. The permeate is withdrawn from the membrane at a constant flow rate *Q_P_* and the feed solution is fed to the membrane at a constant flow rate *Q_F_*. For concentration alone, there is no diafiltration water (*Q_W_* = 0). Equation (1) gives the final mixing-cup retentate concentration <*C_R_*> [15]:

(1) $$\ln\frac{\langle C_{R}\rangle }{C_{F}}=\left(1-\hat{S_{O}}\right)\ln VCF,$$

where the volume concentration factor *VCF* = *V_F_*/*V_R_*, and the lumped sieving coefficient ($\hat{S_{O}}$) is defined by Equation (2) [14]:

(2) $$\hat{S_{O}}=\frac{C_{P}}{\langle C_{R}\rangle }=\frac{S_{O}}{1-\hat{Q}\left(1-S_{O}\right)}.$$

Equation (3) defines the dimensionless permeate flow rate $\hat{Q}$:

(3) $$\hat{Q}=\frac{Q_{P}}{Q_{F}}.$$

Equation (4) gives the mixing-cup permeate concentration <*C_P_*> by mass-balance [14]:

(4) $$\langle C_{P}\rangle \left(VCF-1\right)=C_{F}VCF-\langle C_{R}\rangle .$$

The feed solution for diafiltration is the final solution from concentration. At the start of constant volume diafiltration, water for diafiltration is added continuously to the feed tank so *Q_W_* = *Q_P_*, and the volume of solution in the feed tank is constant. The number of diafiltration volumes, *N_D_*, of water added is *N_D_* = *Q_P_t*/*V_F_*. Equation (5) gives the mixing-cup retentate concentration, <*C_R_*> [14]:

(5) $$ln\left(\frac{\langle C_{R}\rangle }{C_{F}}\right)=-N_{D}\hat{S_{O}}.$$

Equation (6) gives the permeate mixing-cup concentration by mass balance:

(6) $$\langle C_{P}\rangle =\frac{C_{F}-\langle C_{R}\rangle }{N_{D}}.$$

Using these models, the composition of the milk protein concentrate product is determined uniquely by two mutually independent model parameters: (1) the protein sieving coefficient, and (2) the processing volumes chosen for concentration and diafiltration.

## 3. Experimental

### 3.1. Membrane Surface Modification

The regenerated cellulose ultrafiltration membranes of molecular weight cut-off (MWCO) 100 kDa (PLCHK) and 300 kDa (PLCMK) were obtained in the Pellicon-2^™^ Mini format (EMD Millipore, Billerica, MA). Membranes were 1000 cm^2^ in area and incorporated suspended screens in the retentate channel (V screen), recommended for feed solutions containing suspended solids or having a high viscosity [18]. The membranes were charge modified following the procedure of Riordan et al. [19] as in our previous work [14,20]. Briefly, hydroxyl groups on the membrane were reacted with allyl glycidyl ether to activate the membranes. The allyl groups were brominated using N-bromosuccinimide (NBS), and then reacted with the amine moiety of taurine to place a negative charge (SO_3_^−^) on the membrane (see Supplementary Materials for chemical structures).

### 3.2. Ultrafiltration Experiments at Total Recycle

Raw skim milk was obtained from the Babcock Hall Dairy Plant, Madison WI and stored at 4 °C, adding 0.05% NaN_3_ to prevent microbial growth. The skim milk had a pH of 6.74 ± 0.05 and a conductivity of 7–8 mS cm^−1^. The skim milk was used “as is” for the ultrafiltration experiments with no pre-filtration.

The sieving coefficients were measured under “total recycle” wherein both permeate and retentate streams were recycled back to the feed tank. Before taking samples for analysis, a steady state was established by the recirculation of 2 L of skim milk at a retentate flow rate of 1275–1350 mL min^−1^ or equivalently 765–810 L m^−2^ h^−1^ (LMH) for 8–10 h at 22 °C while permeate was recycled back to the feed tank at a lower flow rate of 15 mL min^−1^ (9 LMH). Permeate flux was increased to the target value, and 10 mL samples were collected from the permeate and retentate tubing for later analysis. The feed stream pressure was 2 bar and the differential pressure on the retentate channel was approximately 0.3 bar, with the inlet pressure maintained using the pinch clamp on the retentate exit.

### 3.3. Concentration Experiments

**Figure 2:**
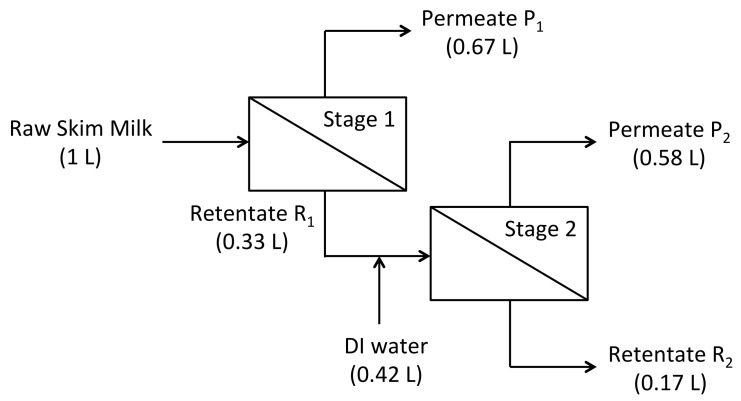
Schematic diagram for milk protein concentrate (MPC) manufactured from skim milk using concentration and diafiltration. The membranes used in Stages 1 and 2 were the same.

An industrial process for producing 80% protein dry-basis MPC was simulated using the 100 kDa unmodified and 100 kDa negatively charged membrane and a batch size of 1 L of skim milk (Figure 2). The membrane area was 1000 cm^2^. The retentate flow rate was 765–810 LMH, and the permeate flow rate was 16 LMH during concentration and 13 LMH during diafiltration. During diafiltration, the viscosity of the retentate increased, causing the membrane inlet pressure to increase from 2 to 2.7 bar. The viscosity of skim milk increases exponentially with concentration. Meyer et al. [17] clearly describe the problem of viscosity increasing during the ultrafiltration of skim milk: shear, pressure, flux, and deposit layer formation are linked in conventional membrane separations. They solved this problem by creating shear using a rotating disc. We did not use this approach. We used membrane modules with suspended feed stream screens, meant especially for viscous, concentrated protein solutions. As a result, the feed stream pressure increased modestly, never exceeding 45 psig (3 bar). Given that we increased the milk concentration by 500%, a 35% increase in pressure is comparatively negligible, and this use of suspended screens solved the problem of viscosity.

Stage 1 was a three-fold volume reduction of the skim milk, generating 0.67 L of permeate, P_1_, and 0.33 L of retentate, R_1_. Stage 2 was a constant volume diafiltration of the retentate R_1_ using 1.26 diafiltration volumes (i.e., 0.42 L) of deionized water, followed by a two-fold volume reduction of the diafiltered retentate from 0.33 L to 0.167 L. The final concentrate, R_2_, was fully recovered using 100 mL of deionized water to displace retentate trapped in the membrane hold-up volume. After the experiment, membranes were cleaned using deionized water, 0.2% Tergazyme^™^ (Alconox, White Plains, NY, USA), 1% *v*/*v* Ultrasil-75^®^ Acid (Ecolab, St. Paul, MN, USA) at pH 2.5 followed by 0.1 M NaOH.

### 3.4. Analysis of Serum Protein

Serum proteins (SP) were analyzed by HPLC (High Performance Liquid Chromatography) using a 1 mL cation exchange column (HiTrap^®^ SPFF, GE Healthcare Bio-Sciences, Uppsala, Sweden). SPs were positively charged at pH 4 and bound to the negatively charged column. Prior to HPLC analysis, caseins were precipitated at pH 5.0, pelleted by centrifugation at 4000× *g* and 0 °C for 1 h, and residual casein in the supernatant removed by 0.45 μm filtration followed by 0.22 µm filtration. The chromatographic procedure consisted of four steps: (1) column equilibration using 10 mM sodium lactate, pH 4 (buffer A); (2) injection of 2 mL of the sample and washing out the unbound sample using buffer A; (3) eluting bound protein using buffer B (buffer A + 1 M NaCl) and (4) column re-equilibration using buffer A. The peak area at 280 nm was used to calculate SP concentration using a calibration curve.

### 3.5. Analysis of True Protein (TP)

Total Kjeldahl nitrogen (TKN) [21] was measured using a digestion system (Foss Tecator 2020, Hoganas, Sweden) and rapid distillation apparatus (RapidStill II, Labconco, Kansas City, MO, USA). Proteins were precipitated using 12% trichloroacetic acid and the supernatant analyzed for non-protein nitrogen (NPN) [21]. True protein (TP) was calculated using the formula 6.38 × (TKN − NPN) [21]. Skim milk contained 5.4 ± 0.1 g L^−1^ SP and 28.1 ± 2.3 g L^−1^ TP (*n* = 5). Casein was 22.8 ± 2.3 g L^−1^, calculated by difference.

### 3.6. Dry Solids

An aluminum weighing dish was loaded with 3 mL of the sample and placed into an oven at 100 °C for 16 h. Dried samples were placed into a desiccator for 30 min to cool, and the wet and dry masses were used to calculate dry solids.

## 4. Results

### 4.1. Sieving Coefficients Using Different Membranes

The sieving coefficients of SP and TP for all four membranes are shown in Table 1. The goal was to operate at a higher permeate flux without exceeding the sieving coefficients of the 30 kDa unmodified polyethersulfone (PES) membrane used in industry. The 100 kDa unmodified membrane failed this test because although the permeate flux was higher than the 30 kDa unmodified membrane, the sieving coefficients were also higher. The 100 kDa negatively charged membrane was successful because the flux of 24 LMH was four-fold higher than the 30 kDa unmodified membrane and the sieving coefficients of SP and TP were not different (*p* > 0.05). A flux excursion with the 100 kDa unmodified membrane and the 100 kDa negatively charged membrane (data not shown) showed that the sieving coefficients did not vary with permeate flux in the range of 6 to 30 LMH (*p* > 0.05). The 300 kDa negatively charged membrane was marginally successful because the flux of 30 LMH was five-fold higher than the 30 kDa unmodified membrane, and while the sieving coefficient of TP was not different, the sieving coefficient of SP was somewhat higher (*p* > 0.05). In conclusion, the negatively charged wide-pore ultrafiltration membranes had a substantially higher permeate flux at essentially the same protein retention as the 30 kDa unmodified membranes used in industry today for MPC manufacturing.

### 4.2. Comparison of Concentration Measurements to Mass Balance Calculations

Experimental measurements of protein concentration were compared to calculations from mass balance models for stage 1 ultrafiltration (Equations (1) and (4)) and stage 2 diafiltration (Equations (5) and (6)) in the manufacturing of 80% protein dry-basis MPC. The purpose of this work was to validate the replacement of trial and error experimentation with mass balance models in the design of new processes. The sieving coefficients measured at total recycle were used in the calculations.

As shown in Table 2, for stage 1 and the 100 kDa unmodified membrane, measured and calculated concentrations for SP in R_1_ were not different (*p* > 0.05), but the measured concentration for P_1_ was slightly lower than the calculated concentration (*p* = 0.03). For stage 2, the measured and calculated concentrations of SP and TP in P_2_ were not different, and no difference was found for TP in R_2_ (*p* > 0.05), but the measured concentration of SP for R_2_ was slightly higher than the calculated concentration (*p* = 0.04).

As shown in Table 3, for stage 1 and the 100 kDa negatively charged membrane, the measured and calculated concentrations of SP and TP in P_1_ and R_1_ were not different (*p* > 0.05). TP for P_1_ was not measured because it contained no casein, making the TP and SP concentrations equal. TP for R_1_ was not measured in order to feed all of that liquid to stage 2. For stage 2, measured and calculated concentrations of SP and TP in P_2_ and R_2_ were not different (*p* > 0.05). In summary, 10 of the 12 comparisons between measured and calculated protein concentrations in stages 1 and 2 were the same, and the other two comparisons were slightly different.

The measured and calculated protein recoveries of SP and TP are shown in Table 4. Protein recovery was defined as the ratio of the mass of protein in the product stream R_2_ to that in the skim milk feed stream. Measured and calculated recoveries of SP and TP were not different for the 100 kDa unmodified membrane (*p* > 0.05), but there was a small difference between the measured and calculated recoveries of SP and TP using the 100 kDa negatively charged membrane (*p* < 0.05). The measured recoveries of SP and TP were not different between the 30 kDa unmodified membrane and the 100 kDa charged membrane (*p* > 0.05).

### 4.3. Non-Protein Permeate Solids

The non-protein permeate solids (NPPS) in the comingled permeate plus diafiltrate (P_1_ + P_2_) consisted of lactose, NPN, ash, and other small molecules. The NPPS was calculated as the difference between total dry solids and protein dry solids, and was (1) 40.9 g L^−1^ for the 30 kDa unmodified membrane (value reported by the Wisconsin Center for Dairy Research); (2) 43.4 ± 0.9 g L^−1^ for the 100 kDa unmodified membrane; and (3) 46.3 ± 0.4 g L^−1^ for the 100 kDa negatively charged membrane (average ± SD, *n* = 2). The NPSS was significantly lower for the 30 kDa unmodified membrane compared to either of the 100 kDa membranes (*p* < 0.05). Thus, the wide-pore membranes permeated small molecules more readily than the 30 kDa unmodified membrane. The NPSS was significantly lower for the 100 kDa unmodified membrane compared to the 100 kDa negatively charged membrane (*p* < 0.05). Thus, the negatively charged wide-pore membranes permeated NPPS more readily than the unmodified wide-pore membrane.

## 5. Discussion

This work is the first to show the advantages of wide-pore charged ultrafiltration membranes for milk protein concentration. This result is important because normally as the pore size of a membrane increases, one trades off increased flux for decreased protein recovery [22]. The principal discovery of the present work is that this trade-off can be overcome using 100 kDa or 300 kDa negatively charged ultrafiltration membranes.

### 5.1. Sieving Coefficients during Total Recycle

Skim milk protein consists of 80% caseins and 20% SP, and together these equal the TP content. Caseins exist as micelles and are retained by all four ultrafiltration membranes tested in this work. Therefore, differences in the sieving coefficients of SP alone caused differences in membrane performance. For example, the sieving coefficients for TP were not different between the 100 kDa and 300 kDa negatively charged membranes, and the 30 kDa unmodified membrane. Compared to the 30 kDa unmodified membrane, the sieving coefficient of SP was not different for the 100 kDa negatively charged membrane, it was 4.6-fold greater for the 300 kDa negatively charged membrane (*p* > 0.05).

The relative protein mass flux through the membrane is the product of the protein sieving coefficient and the permeate flux. Compared to the 30 kDa unmodified membrane, the permeate flux was four-fold greater for the 100 kDa negatively charged membrane, and five-fold greater for the 300 kDa negatively charged membrane. The tradeoff between the 100 kDa and 300 kDa negatively charged membranes is that one must balance higher flux (four-fold versus five-fold) against slightly higher losses of SP (*S_O_* = 0.014 versus *S_O_* = 0.046). There was no tradeoff in terms of losses of TP, because both of the negatively charged membranes matched the sieving coefficients for the TP of the 30 kDa unmodified membrane used currently in the dairy industry for the manufacturing of milk protein concentrate, but at a considerably higher permeate flux.

### 5.2. Concentration and Diafiltration Mass Balance Models

The agreement between the model-calculated and experimentally observed concentrations was remarkable. When comparing experimental observations versus model calculations, six of eight concentrations for SP, and four of four concentrations for TP were identical. This discovery is useful because the model uses only sieving coefficients measured at total recycle that are easy to measure, and are not tied to implementing a specific flow diagram or separation process. Calculating the performance of a multi-stage membrane separation using sieving coefficients measured at total recycle is analogous to calculating the performance of multi-stage distillation using the relative volatility in the McCabe–Thiele model [15]. This allows for the elimination of the expensive and time-consuming experimental trial-and-error approach for membrane system design and operation.

### 5.3. Wastewater Generation and Water Consumption

The goal of ultrafiltration and diafiltration in whey processing is to increase the protein-to-dry-solids ratio in the retentate. Ultrafiltration alone can increase the retentate protein concentration, but it cannot decrease the concentration of small molecules such as lactose, non-protein nitrogen and ash. That requires diafiltration. Water is added during diafiltration to wash out small molecules while retaining protein. The 100 kDa charged membrane had a 13% higher transmission of small molecules than the 30 kDa unmodified membrane at the same protein retention. The impact of this result can be estimated using constant values of *C_F_* and <*C_R_*> in Equation (6). When <*C_P_*> is larger, then *N_D_* is smaller. In other words, less diafiltration water (*N_D_*) is needed to do the same job (constant *C_F_* and <*C_R_*>), when the observed value of <*C_P_*> is larger. Since <*C_P_*> was 1.13 times larger for the 100 kDa charged membrane compared to the 30 kDa unmodified membrane, then *N_D_* can be 1.13 times smaller. This means that compared to the unmodified membrane, the charged membrane consumes less water, and generates less permeate and less wastewater.

## 6. Conclusions

This work made several new advances in the use of wide-pore negatively charged tangential-flow membranes for MPC manufacturing. First, the speed of MPC processing can be increased four-fold without increasing protein losses by using charged membranes. Second, using wide pore charged membranes and permeate flux control made at least a six-fold concentration of skim milk possible. As MPC is sold as a dry powder, essentially all water must be removed eventually. Greater concentration by membranes means less water must be removed downstream in the thermal processes such as evaporation and spray drying. Third, wide pore charged membranes use less water for diafiltration, generating less wastewater. This is because small molecules such as lactose permeate the wide pores of the 100 kDa charged membrane more readily than the tight pores of the 30 kDa unmodified membranes used currently in industry. Fourth, a unique mass balance model was applied that accurately predicted the experimental results, making it possible to replace expensive and time-consuming experiments with computer calculations in the design, operation, and optimization of MPC processes. These novel aspects of the present work are significant to the dairy industry.

## Figures and Tables

**Table 1:** Sieving coefficients (*S_O_*) for serum protein (SP) and true protein (TP) measured at total recycle during the ultrafiltration of skim milk at 22 °C using different membranes.

Membrane	Permeate Flux (L m^−2^ h^−1^, LMH)	*S_O_* SP	*S_O_* TP
30 kDa unmodified	6	0.010 *	0.006 *
100 kDa unmodified	8	0.20 ± 0.02 †	0.036 ± 0.009
	13	0.19 ± 0.02	0.036 ± 0.005
	20	0.16 ± 0.02	0.030 ± 0.003
100 kDa charged	24	0.014 ± 0.003	0.004 ± 0.001
300 kDa charged	30	0.046 ± 0.002	0.008 ± 0.001

* Data provided by the Wisconsin Center for Dairy Research. † Average ± SD, *n* = 2, where *n* is the number of replicates performed using that membrane.

**Table 2:** The calculated and measured mixing-cup concentrations (g L^−1^) of serum protein (SP) and true protein (TP) for the 100 kDa unmodified membrane during the 6-fold volume concentration of skim milk. The streams are feed, first stage permeate (P_1_) and retentate (R_1_), and second stage permeate (P_2_) and retentate (R_2_).

Stream	SP Measured	SP Calculated	TP Measured	TP Calculated
Feed	5.33 ± 0.01	---	29.8 ± 0.5	---
P_1_	0.9 ± 0.1	1.6 ± 0.2	---	1.6 ± 0.2
R_1_	14 ± 1	12.99 ± 0.05	---	88 ± 4
P_2_	1.7 ± 0.2	2.4 ± 0.2	1.7 ± 0.2	2.4 ± 0.2
R_2_	20.6 ± 0.4	17.4 ± 0.8	141 ± 4	167 ± 23

**Table 3:** Calculated and measured mixing-cup concentrations (g L^−1^) of serum protein (SP) and true protein (TP) for the 100 kDa charged membrane during the six-fold volume concentration of skim milk. The streams are feed, first stage permeate (P_1_) and retentate (R_1_), and second stage permeate (P_2_) and retentate (R_2_).

Stream	SP Measured	SP Calculated	TP Measured	TP Calculated
Feed	5.4 ± 0.2	---	25.7 ± 0.7	---
P_1_	0.11 ± 0.04	0.17 ± 0.04	---	0.17 ± 0.04
R_1_	18 ± 1	16.1 ± 0.7	---	78 ± 8
P_2_	0.19 ± 0.03	0.33 ± 0.07	0.19 ± 0.03	0.33 ± 0.07
R_2_	27.7 ± 0.6	25.5 ± 0.7	136 ± 3	128 ± 3

**Table 4:** Measured versus calculated percentage recovery of serum protein (SP) and true protein (TP) in the product retentate (R_2_) for the manufacture of MPC from raw skim milk by ultrafiltration and diafiltration.

Membrane	SP Measured	SP Calculated	TP Measured	TP Calculated
30 kDa unmodified *	98.1	---	99.6	---
100 kDa unmodified	68.1 ± 5.0 †	53.6 ± 4.6	89.3 ± 1.2	91.7 ± 0.7
100 kDa charged	98.9 ± 0.5	94.4 ± 1.1	102.3 ± 0.1	98.8 ± 0.3

* Data provided by the Wisconsin Center for Dairy Research. † Average ± SD, *n* = 2, where *n* is the number of replicate experiments.

## Supplementary Materials

The following is available online at http://www.mdpi.com/2304-8158/7/9/134/s1: Supplement for Structures.
