# Supplementary figures and images for: Milk Protein Concentration Using Negatively Charged Ultrafiltration Membranes
Source: Foods. 2018 Aug 28;7(9):134. doi: 10.3390/foods7090134 (PMC6165199; doi:10.3390/foods7090134)

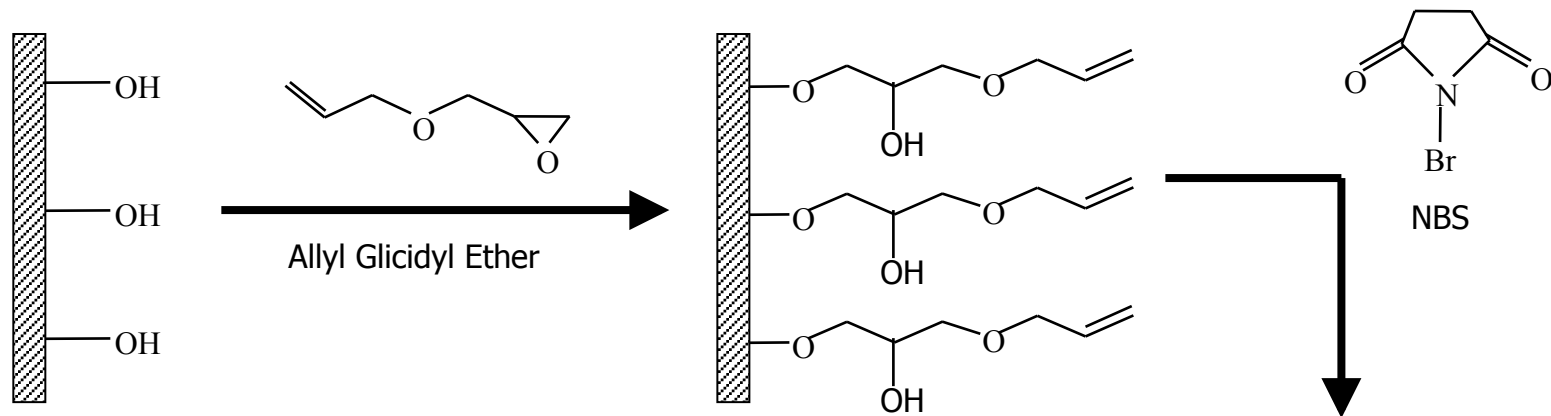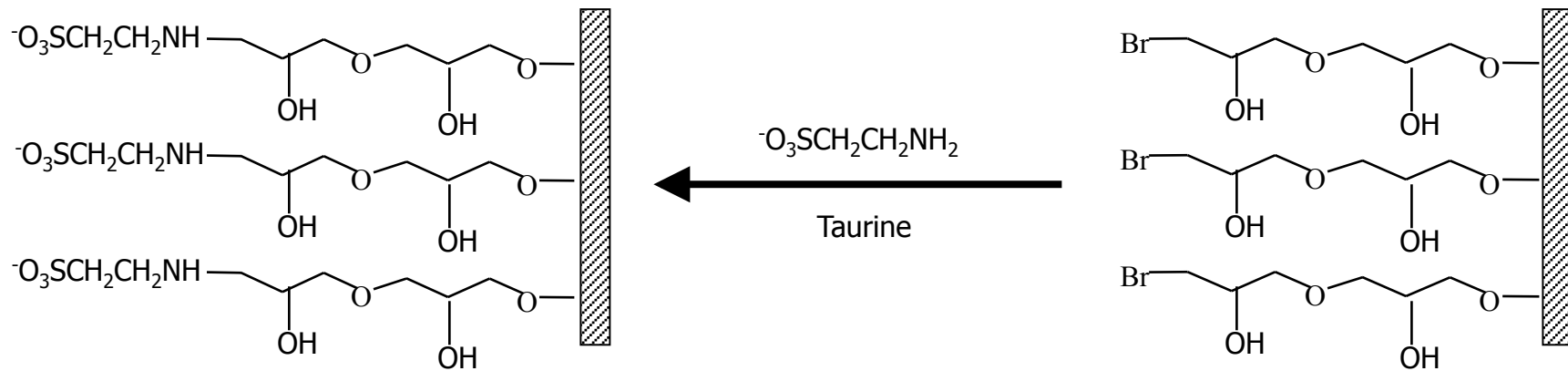

Supplement: Supplementary file 1 [file foods-07-00134-s001.pdf]
